# Effects of Two Different Neuromuscular Training Protocols on Regional Bone Mass in Postmenopausal Women: A Randomized Controlled Trial

**DOI:** 10.3389/fphys.2019.00846

**Published:** 2019-07-10

**Authors:** Elena Marín-Cascales, Jacobo Á. Rubio-Arias, Pedro E. Alcaraz

**Affiliations:** ^1^Research Center for High Performance Sport, Catholic University of Murcia, Murcia, Spain; ^2^Faculty of Sport Sciences, Catholic University of Murcia, Murcia, Spain

**Keywords:** vibration, combined training, bone density, osteoporosis, exercise, menopause

## Abstract

**Background:** Osteoporosis is a condition associated with a greater incidence of fractures, and one of the main health-related concerns in postmenopausal women. To counteract possible reductions in bone properties, physical exercise has been proposed as an effective strategy. Particularly, training interventions with a high osteogenic potential are recommended.

**Purpose:** To analyze the effect of 24 weeks of whole-body vibration and multi-component training on lumbar spine and femoral neck bone mass, and to determine what type of training produces greater adaptations in postmenopausal women.

**Methods:** A total of 38 women completed the study (Clinical Gov database ID: NCT01966562). Participants were randomly assigned to one of the study groups: whole-body vibration group (WBVG), multi-component training group (MTG), or control group (CG). The experimental groups performed a progressive 24-week training (3 sessions/week) program. Bone mineral density (BMD) and bone mineral content (BMC) at the lumbar spine and femoral neck were assessed by Dual-energy X-ray absorptiometry.

**Results:** Significantly and clinically relevant increases in lumbar spine bone mass (BMD: *F* = 3.29; *p* = 0.03; +5.15%; BMC: *F* = 2.90; *p* = 0.05; +10.58%) were observed in WBVG. MTG showed clinically important pre-post-changes on lumbar spine BMC (+7.78%), although there was no statistical significance (*F* = 1.97; *p* = 0.14). At the femoral neck, no statistically significant increases on bone mass were obtained in either training group. No changes were obtained in any variable in the CG. Additionally, no statistically significant differences were found between groups.

**Conclusion:** The results indicated that 24 weeks of supervised WBV and MT may counteract the rapid loss of bone mass after the cessation of menstruation, thus improving postmenopausal women bone health. However, in the absence of statistically significant differences between groups, it is not possible to determine which training protocol produces greater adaptations.

**Clinical Trial Registration:** www.ClinicalTrialsgov, identifier: NCT01966562.

## Introduction

With the global population life expectancy increasing over the years, a higher prevalence of chronic diseases in the elderly has been observed (United Nations, 2013). Illnesses like osteoporosis, which are associated with greater incidence of fractures, are one of the main health-related concerns that can result in important health problems (Kanis et al., 2008). The estimated number of osteoporotic fractures is approximately 9.0 million worldwide per year, of which 61% are women (Johnell and Kanis, 2006). The most common osteoporotic fractures occur on the proximal femur, spine and wrist (Cummings and Melton, 2002). Hip fracture is an important consequence of low bone mineral density (BMD) and leads to a larger physical incapacity compared with all other types of fracture (Cummings and Melton, 2002), with an estimated incidence of 2.6 million by the 2025 (Gullberg et al., 1997). The loss of estrogen’s protective effects on skeletal structure after menopausal transition intensifies the decline in bone density, which is a result of a sudden imbalance between resorption and new bone formation (Christenson et al., 2012). Said instability is thought to occur due to hormonal changes that take place during the post-menopause period (Yuen et al., 2010; Gomez-Cabello et al., 2012).

To counteract this issue, physical exercise has been widely used as an effective lifestyle approach to improve and maintain bone properties, positively affecting quality of life (Gomez-Cabello et al., 2012; McMillan et al., 2017). The theoretical background for this approach is provided by the mechanostat theory (Frost, 1987), which proposes that bones adapt their structure in response to the strain caused mainly by physiological loads (i.e., muscle contractions). If the strain imposed is below the minimum effective load, no changes on bone structure are to be expected (Tyrovola, 2015). In contrast, when a sufficient mechanical stimulus is imposed, the bone adjusts in order to reduce subsequent strains under similar loads, or those below the lower limit of the minimum effective strain (Tyrovola, 2015). Therefore, exercise-based interventions that reinforce muscular contractions are important strategies to be used with post-menopausal women.

Along these lines, traditional resistance strength training has been shown to increase BMD levels at the spine and femoral neck because of the weight imposed on the skeleton during the training sessions (Mosti et al., 2013). However, the effect on bone in response to training is different between age groups and sex. In fact, Bassey et al. (1998) compared the effects of jumping exercise on BMD in premenopausal and postmenopausal women, presenting statistically significant increases in femoral neck bone mass after 5 months of this type of intervention in premenopausal women, whereas no changes were observed in postmenopausal women. Another study examining sex differences on bone mass showed that 24 weeks of high-intensity, free-weight exercise program increased spine and trochanter BMD in healthy older men but not in women (Ryan et al., 2004), suggesting that women may have needed more long-term training to obtain a statistically significant increase in bone mass (Ryan et al., 2004). These studies indicate that women, particularly after the menopause period, attain less improvement on bone mass and highlight the need to conduct further research on how bone adapts to resistance training in postmenopausal women.

Most recently, Multi-component training (MT) has emerged as a training method aimed, in particular, at developing bone mass (Marques et al., 2011; Gianoudis et al., 2014; Marin-Cascales et al., 2017a). This method consists of combining different exercise modalities in the same workout. Previous studies addressing MT have shown that coupling high-impact aerobic activities with resistance strength training is effective in eliciting or maintaining bone mass in older adults (Kemmler et al., 2010; Gomez-Cabello et al., 2012), due to its potential to generate important osteogenic adaptations (as a response to mechanical stimulus) (Guadalupe-Grau et al., 2009). However, there is no agreement in the literature regarding whether MT results in increments in bone mass in postmenopausal women (Martyn-St James and Carroll, 2009; Marin-Cascales et al., 2015, 2017b). In fact, Tolomio et al. (2010) demonstrated enhanced femoral neck T-score and physical function capacity in postmenopausal women following 11 months of a multi-component dual-modality exercise program, which included combined resistance training, aerobic exercise, balance, and joint mobility activities. Multanen et al. (2014) found an improvement in bone mineral content (BMC) at the femoral neck, but no changes were observed in the lumbar spine, after 12 months of aerobic training combined with step-aerobic jumping exercises.

Another neuromuscular training program designed to increase bone mass that has received attention in recent years is Whole-body vibration training (WBV), which consists of a mechanical stimulus that is characterized by oscillatory action (Rauch et al., 2010). In WBV, the subject maintains a standing static position or performing dynamic exercises on a platform that delivers a vibrating stimulus through the kinematic chain of the body to the muscles and bones (Cheung and Giangregorio, 2012). The skeletal system’s response to WBV results in an anabolic effect on bone tissue, specifically increasing bone mass following this type of training (Von Stengel et al., 2011). It is known that vibration training produces micro trauma to the bone, which is later restored by the osteoblast cells (Burr et al., 1985). Some studies have examined the effects of WBV on bone mineral levels (Gomez-Cabello et al., 2014; Marin-Cascales et al., 2015; Fratini et al., 2016). Zaki (2014) conducted a study using 8 months of WBV (10 sets of 1 min vibration and a frequency of 16 Hz, 3 times per week) and showed statistically significant improvements in bone mass at the lumbar spine in obese postmenopausal women after training. In contrast, a meta-analysis by Slatkovska et al. (2010) observed a statistically significant positive effect of WBV on BMD of the hip but not of the lumbar spine. Therefore, it is necessary to develop more training methods with specific recommendations to prevent osteoporosis.

Considering the controversy with regard to osteogenic potential of these training protocols in postmenopausal women and given the femoral neck and lumbar spine are two of the most sensitive areas of fracture, the aims of this study were: (1) to analyze the effect of 24 weeks of WBV and MT on BMD and BMC of the lumbar spine and femoral neck, and (2) to determine what type of training produces greater adaptations in this population. It was hypothesized that both training protocols would be effective methods to improve bone mass in the abovementioned sites, and that MT would present a higher osteogenic potencial due to the high-impact (and strain-inducing) activities.

## Materials and Methods

### Experimental Design

A randomized controlled trial of 24 weeks, intra and inter-participants design with pre, and post-test, with control group was conducted. Participants were matched by BMD and were randomly assigned to one of three groups: Whole-body vibration group (WBVG) (*n* = 25), Multi-component training group (MTG) (*n* = 25), and control group (CG) (*n* = 15). Women in CG did not participate in any exercise program during the intervention period and performed only pre- and post-test at 24 weeks. Thus, more subjects were allocated to the intervention groups, as these could potentially have higher dropout rates. Block size randomization was ensured by using the Software Research Randomizer (Version 4.0, Lancaster, PA, United States) to randomize subjects. To ensure allocation concealment, a member of our research group, not directly involved in this project, was responsible for the randomization of the sample (Dettori, 2010).

### Subjects

Sixty-five postmenopausal women volunteered to participate and were recruited by non-probability convenience sampling. Participants were included in the study if they had reached menopause at least 3 years before the beginning of the experimental procedures. Women were excluded from the study if they: (1) presented a high level of osteoporosis (BMD < 70 g/cm^2^); (2) were taking any drugs or supplementation (including calcium and vitamin D) that could affect bone structure or the neuromuscular system; (3) had orthopedic prosthetic implants in the lower limbs and / or spine; (4) had herniated discs; (5) had suffered ocular diseases that affected the retina; (6) had suffered severe cardiovascular diseases; (7) had epilepsy; (8) had a pacemaker or osteosynthesis implant; (9) practiced more than 150 min of moderate-intensity exercise per week (American College of Sports Medicine, 2013); and (10) had a lower than 90% attendance to the stipulated program. All volunteers gave written informed consent to participate in the study prior to inclusion and were instructed to maintain their normal daily routines and eating habits. The study protocol was approved by the Human Ethics Committee of the Catholic University of Murcia (UCAM), number CE111212. At the end of the study, a total of 38 women (58.5%) completed the program: WBVG (*n* = 15), MTG (*n* = 13), and CG (*n* = 10). The participant characteristics are given in Table 1.

**Table 1:** Descriptive data of each group.

Variable	WBVG (*n* = 15)	MTG (*n* = 13)	CG (*n* = 10)	Total (*n* = 38)	*p*
Age (years)	59.6 ± 5.9	58.0 ± 7.3	62.4 ± 5.1	59.8 ± 6.3	0.62
Height (cm)	156.8 ± 5.0	155.0 ± 4.6	157.4 ± 4.2	156.4 ± 4.7	0.68
Body mass (kg)	77.2 ± 13.5	71.5 ± 9.8	72.7 ± 10.1	74.0 ± 11.5	0.64
BMI (kg⋅m^-2^)	31.4 ± 5.7	29.7 ± 3.7	29.4 ± 4.8	30.3 ± 4.8	0.44

*Values are given as mean ± SD. BMI, body mass index; CG, control group; MTG, multi-component training group; WBVG, whole-body vibration group. *p* = *p*-value from the Levene test.*

### Bone Measurements

The BMD (g cm^2^) and BMC (g) measurements were determined before the start of training sessions and at 24 weeks of treatment. All tests were conducted in the University’s research center and administered by the same investigator, who was blinded to the group assignment. Lumbar spine (L1-L4) and left femoral neck were assessed by dual-energy X-ray absorpiometry (DXA) (XR-46, Norland Corp., Fort Atkinson, WI, United States), using standard protocols. Participants were placed in the supine position on the table. Prior to each testing session, the DXA scanner was calibrated using a lumbar spine phantom. The bone data were obtained by manual method, according to the recommendations outlined by the manufacturer. The intraclass correlation coefficient was excellent (ICC = 0.89; CI = 95%).

### Training

Participants in WBVG and MTG completed 3 training sessions per week (in non-consecutive days) for 24 weeks. The total training volume was 72 sessions. Each session started with a specific warm-up, consisting of 8 min cycling on a stationary bike at moderate intensity, followed by active stretching and joint mobility exercises of the lower limbs. All training sessions were completed in an indoor facility (temperature: 21–23°C, humidity: 57–61%) and were supervised by the same specialized and certified trainer (NSCA-CPT). The characteristics and the progression of training programs are presented in Table 2.

**Table 2:** Characteristics of training programs.

	M 1	M 2	M 3	M 4	M 5	M 6
	(Weeks 1–4)	(Weeks 5–8)	(Weeks 9–12)	(Weeks 13–16)	(Weeks 17–20)	(Weeks 21–24)
**Whole body vibration training**						
Sets per session	5–6	6–7	7–8	8–9	9–10	10–11
Working time (min)	45s–1	1	1	1	1	1
Frequency (Hz)	35	35	35	40	40	40
Amplitude (mm)	4	4	4	4	4	4
Recovery time (min)	1	1	1	1	1	1
**Multi-component Training**						
Height of drop jump (cm)	–	5	10	15	20	25
Walking time (min)	35	40	45	50	55	60
Maximum Heart Rate Reserve (%)	50	55	60	65	70	75

*M, mesocycle.*

#### Whole-Body Vibration Training

The participants in the WBVG performed the vibration training protocol on a sinusoidal vertical vibration platform (Power Plate Next Generation; Power Plate North America, Northbrook, IL, United States). During each session, the participants stood with their feet positioned side-by-side on the platform and the flexion angle of the knee and hip was set at 120° (Hazell et al., 2007). The arms were crossed and the shoulder flexed at 90° to maintain the arms parallel to the floor. The WBVG performed dynamic calf raises by lifting their heels maximally from the platform (Marín and Rhea, 2010; Bush et al., 2015; Marin-Cascales et al., 2015, 2017b). A metronome was used to control the rhythm of the exercises using a frequency of 100 b.p.m.: 1 b.p.m. for the concentric phase and 5 b.p.m. for the eccentric phase. During the first 2 weeks of training, the WBVG performed 5 sets of 45 s vibration. The training volume progressively increased by incrementing the number of series per session (adding 1 or 2 sets every 4 weeks, until 11 sets were reached). The resting period between sets was 60 s (Cormie et al., 2006). The amplitude (4 mm) and working time (60 s) parameters remained constant for the 24 weeks of training. The training load was increased by increasing the frequency (35–40 Hz) (Marín and Rhea, 2010; Marin-Cascales et al., 2015, 2017b).

#### Multi-Component Training

The MT protocol combined drop jumps and aerobic activity (Guadalupe-Grau et al., 2009; Marin-Cascales et al., 2015, 2017a,b). During the first 4 weeks of training, small reactive vertical jumps (minimizing knee and ankle dorsiflexion) were performed. For the remaining 60 sessions, participants executed drop jumps starting at a height of 5 cm in mesocycle 2 and progressing up to a height of 25 cm in mesocycle 6. The same 4-week progression (week 1: 4 × 10 jumps; week 2: 5 × 10 jumps; week 3: 6 × 10 jumps; week 4: 4 × 10 jumps) was repeated in each mesocycle. Therefore, the number of drop jumps was the same every 4 weeks, and an undulatory progression was used (the volume increased during 3 weeks and decreased during 1 week). The aerobic exercise was performed after the drop jumps (McCarthy et al., 1995). The volume increased linearly over the 24 weeks. Participants walked at an intensity range between 50 and 75% of maximum heart rate reserve and the minutes ranged between 30 and 60 min. The gait speed used to establish the exercise intensity was determined prior to initial testing using a computer/heart rate monitor.

### Statistical Analyses

Statistical analysis was performed with the Statistical Package for the Social Sciences (SPSS, version 19, SPSS Inc, Chicago, IL, United States) for Windows. A descriptive analysis was conducted to detail and analyze the characteristics of the sample participating in the study. Chi-square statistic was performed to determine if there were statistically significant differences in the number of participants in each intervention group. Homogeneity of variance was investigated using the Levene test. To examine potential between-group baseline differences, a one-way ANOVA was applied.

For the inferential analysis, a Kolmogorov–Smirnov test was performed to establish the normality of sampling distribution and analysis of runs to observe the independence of observations. To examine the effects of the interventions on BMD and BMC at the femoral neck and lumbar spine, three by two (group by time) ANOVA’s with repeated measures on one factor (time) were conducted. If there was a statistically significant effect of time (*p* ≤ 0.05), then an ANOVA test was performed to determine where the statistically significant differences occurred for each group. If there was a statistically significant interaction effect (time × group) (*p* ≤ 0.05), a Tukey *post hoc* test was conducted to determine where the statistically significant differences occurred between groups. Percentage change from pre- to post-test was calculated for BMD and BMC for each group, and the effects of the interventions were evaluated for clinical importance. An effect was considered relevant when the percentage change was greater than the minimum clinically important change (MCIC). The MCIC was calculated using a distribution-based method as a Cohen’s *d* 0.2 of the between-subject standard deviation (SD) of each variable (Cohen, 1988).

## Results

**FIGURE 1:**
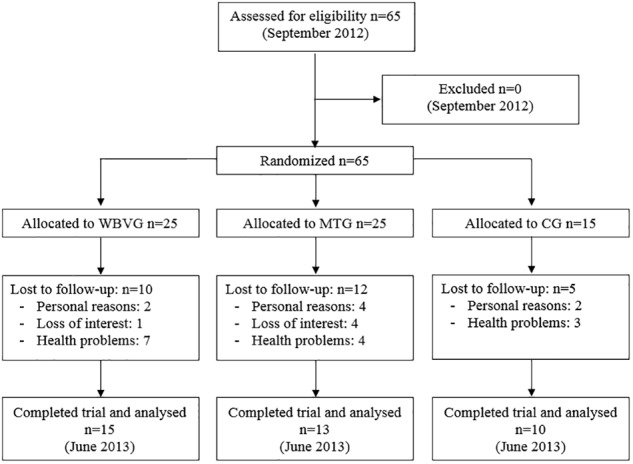
Trial profile. WBVG, whole-body vibration group; MTG, multi-component training group; CG, control group.

Figure 1 shows the flow diagram of the participants in the trial. Twenty-seven women withdrew (41.5%) from training during the course of the study (40% for WBVG, 48% for MTG, and 33% for CG). None of the drop-outs left the program as a result of injuries or adverse responses to the treatment. Regarding the compliance level of the intervention, participants who were included in the final analysis attended the 96.5% of the total number of training sessions (WBVG = 97.2% and MTG = 95.8%).

No statistically significant differences were detected in terms of number of participants among groups. Baseline values for bone parameters did not significantly differ between groups (Table 3).

**Table 3:** Baseline values for lumbar spine and femoral neck BMD and BMC for each group.

Variable		WBVG	MTG	CG	*p*
Lumbar Spine	BMD (g/cm^-2^)	0.97 ± 0.17	0.97 ± 0.22	0.93 ± 0.16	0.81
	BMC (g)	55.22 ± 9.03	58.23 ± 14.09	52.70 ± 11.42	0.53
Femoral Neck	BMD (g/cm^-2^)	0.82 ± 0.17	0.89 ± 0.12	0.85 ± 0.10	0.40
	BMC (g)	2.43 ± 0.45	2.59 ± 0.34	2.49 ± 0.33	0.61

*Data are given as mean ± SD. BMD, bone mineral density; BMC, bone mineral content; CG, control group; MTG, multi-component training group; WBVG, whole-body vibration group. *p* = *p*-value from the baseline One-way ANOVA.*

### Bone Outcomes

**FIGURE 2:**
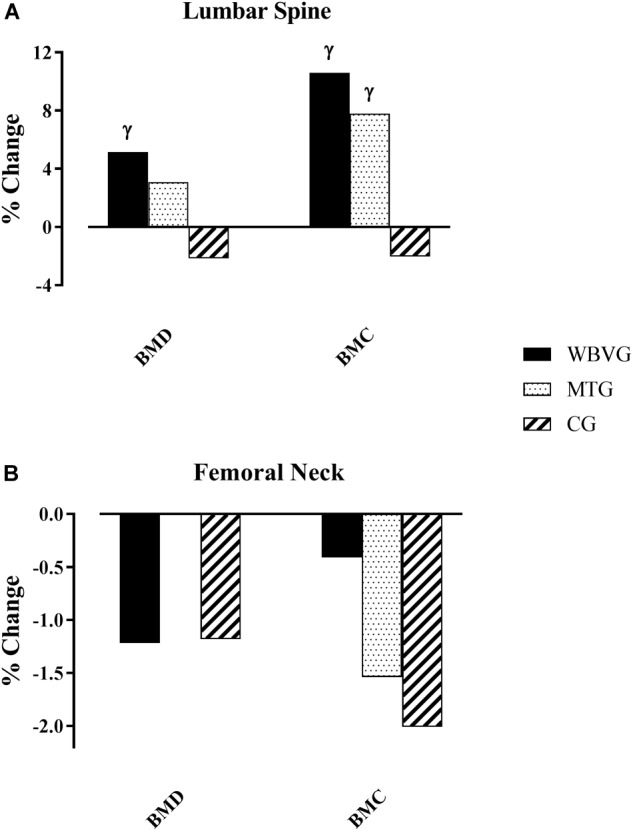
Percentage change in bone parameters. **(A)** Lumbar spine; **(B)** femoral neck. ^γ^Clinically relevant changes. WBVG, whole-body vibration group; MTG, multi-component training group; CG, control group.

The bone parameters changes are shown as mean ± SD (Table 4) and percentages (Figure 2). The vibration treatment promoted statistically significant and clinically relevant increases in lumbar spine BMD (WBVG: *F* = 3.29; *p* = 0.03; +5.15%) and BMC (WBVG: *F* = 2.90; *p* = 0.05; +10.58%) after 24 weeks. Regarding MTG, the percentage change was clinically important in BMC (+7.78%), although there were no statistically significant pre-post differences (*F* = 1.97; *p* = 0.14). In lumbar spine BMD, no statistically significant or clinical relevant changes were obtained (*F* = 1.60; *p* = 0.21; +3.09%) following this protocol.

**Table 4:** Changes in bone parameters.

			PRE	POST	MD (I-J) (95% CI)	*p*
WBVG	Lumbar spine	BMD (g/cm^-2^)	0.97 ± 0.17	1.02 ± 0.20	–0.051 (-0.097; -0.004)	0.03^∗^
		BMC (g)	55.22 ± 9.03	61.06 ± 19.28	–5.842 (-11.736; 0.051)	0.05^∗^
	Femoral neck	BMD (g/cm^-2^)	0.82 ± 0.17	0.81 ± 0.15	0.010 (-0.015; 0.034)	0.43
		BMC (g)	2.43 ± 0.45	2.42 ± 0.38	0.013 (-0.060; 0.086)	0.72
MTG	Lumbar spine	BMD (g/cm^-2^)	0.97 ± 0.22	1.00 ± 0.21	–0.030 (-0.078; 0.018)	0.21
		BMC (g)	58.23 ± 14.09	62.76 ± 12.99	–4.539 (-10.655; 1.577)	0.14
	Femoral neck	BMD (g/cm^-2^)	0.89 ± 0.12	0.89 ± 0.13	0.005 (-0.020; 0.030)	0.69
		BMC (g)	2.59 ± 0.34	2.55 ± 0.37	0.037 (-0.039; 0.113)	0.33
CG	Lumbar spine	BMD (g/cm^-2^)	0.93 ± 0.16	0.91 ± 0.19	0.018 (-0.037; 0.073)	0.51
		BMC (g)	52.70 ± 11.42	51.63 ± 11.06	1.066 (-5.907; 8.039)	0.76
	Femoral neck	BMD (g/cm^-2^)	0.85 ± 0.10	0.84 ± 0.11	0.015 (-0.014; 0.044)	0.29
		BMC (g)	2.49 ± 0.33	2.44 ± 0.35	0.044 (-0.042; 0.130)	0.31

*Data are presented as mean ± SD. BMC, bone mineral content; BMD, bone mineral density; CI, confidence interval; CG, control group; MD (I-J), mean difference (pre-post); MTG, multi-component training group; WBVG, whole-body vibration group. ^∗^Statistically significant difference from pre- to post-training (*p* ≤ 0.05).*

Concerning femoral neck, no statistically significant or clinically relevant effects were found on BMD (WBVG: *F* = 0.93; *p* = 0.43; -1.22%; and MTG: *F* = 0.49; *p* = 0.69; 0%) and BMC (WBVG: *F* = 0.45; *p* = 0.72; -0.41%; and MTG: *F* = 1.19; *p* = 0.33; -1.54%) when compared with baseline values (Table 4) in either training group.

No statistically significant or clinically relevant pre- and post-test changes were observed in the CG for any of the bone measurements. There were no statistically significant differences in bone parameters at lumbar spine and femoral neck between groups.

## Discussion

This study was conducted to determine the effects of 24 weeks of two different training protocols, WBV and MT, on lumbar spine and femoral neck bone mass in postmenopausal women. The main findings indicate that WBV was efficacious in increasing bone mass at the lumbar spine and that MT presented clinically relevant changes in BMC, even though no statistically significance was found with this protocol. The post-intervention values were not significantly different at this site when comparing both exercise groups with control participants, despite the reduction of 2.15 and 2.03% in BMD and BMC, respectively, in the CG. Regarding femoral neck bone mass, pre-post changes were not statistically significant or clinically important in both training interventions. Again, no statistically significant differences were found between experimental and control groups.

### Whole-Body Vibration Training

The present study shows that BMD and BMC at the lumbar spine significantly and relevantly increased following WBV (5–11 min; 35–40 Hz; 4 mm; 3 days per week). This is in accordance with the data presented by Lai et al. (2013) that reported increments of lumbar spine BMD by 2.0% with 24 weeks of WBV (30 Hz; 3.2 *g*; 3 times per week; in a natural full-standing posture for 5 min) in postmenopausal women. In addition, Beck and Norling (2010) observed that a lower intensity WBV (15 min; 30 Hz; 0.3 *g*) or a higher intensity (6 min; 12.5 Hz; 1 *g*), which only included static exercises twice-weekly, maintained bone mass parameters compared with controls that showed bone loss at the trochanter BMC (-6%, *p* = 0.03) and lumbar spine BMC (-6.6%, *p* = 0.02). However, Bemben et al. (2010) demonstrated no changes in lumbar spine, proximal femur and forearm bone metabolism following an 8-month program of WBV (1–3 sets of 15 to 60 s; 30–40 Hz, 2–4 mm peak to peak) using dynamic movements during vibration combined with resistance training in postmenopausal women. When comparing WBV protocols, it seems that the improvements in bone mass may be associated with longer sessions’ duration. It is suggested that the interventions with a cumulative dose (total time in which the participants stand on vibration plate) over 1000 min were correlated to positive results in BMD (Fratini et al., 2016).

Regarding femoral neck BMD and BMC, no statistically significant or clinically relevant differences were obtained after WBV. Nevertheless, some studies have found significant improvements with WBV (Verschueren et al., 2004; Ruan et al., 2008). Verschueren et al. (2004) observed the effects of a WBV program using static and dynamic knee-extensors exercises (35–40 Hz; 2.28–5.09 *g*) and found that hip BMD significantly increased by 0.9%. Rubin et al. (2004) reported a 2.2 and 1.5% increase of BMD at the femoral neck and at the spine, respectively, in postmenopausal women following a 13-month WBV (30 Hz; 0.2 g; two 10 min treatments/day; 86% compliance). In our study, after WBV, lumbar spine BMD and BMC increased, which did not occur in the femoral neck. Several studies have shown that the effectiveness of WBV on BMD depends on a combination of variables, which could explain the discrepancies in the literature. For example, it appears that the mechanotransduction may have a differing effect among the body regions because of the non-linearity of the musculoskeletal system and the different body positions used on the vibration platform (Rubin et al., 2003; Kiiski et al., 2008). The absence of improvement in femoral neck BMD in the current study may be explained by the upright posture of the body in which the vibration is conducted along the body’s longitudinal axis. While the lumbar spine is aligned with the direction of the transmitted vibration, in the case of the femoral neck, the vibration transmitted is received at a given angle. Thus, the lumbar site likely received a greater vibration stimulus compared with the femoral neck, thereby achieving a stronger effect on the bone cells (Ruan et al., 2008). When applying WBV programs it seems that higher vibration frequency, between 20 and 50 Hz, provides a more intense training stimulus that can adequately transfer energy to the spine (Lai et al., 2013) and hip (Rubin et al., 2003; Ruan et al., 2008).

### Multi-Component Training

In relation to MTG, our findings illustrate positive, clinically important changes in lumbar spine BMC (+7.78%), although no statistically significant pre-post improvements were found at this location following 24 weeks (BMD: *F* = 1.60; *p* = 0.21; BMC: *F* = 1.97; *p* = 0.14). Nevertheless, lumbar spine BMD was maintained, which can be considered a beneficial outcome for this type of population. Several studies have examined whether MT would increase bone mass and ameliorate osteoporosis in postmenopausal women. Marques et al. (2011) indicated that 32 weeks of progressive MT (2 sessions/week), consisting of weight-bearing activities (marching in place, stepping exercise, and heel-drops), muscular endurance, balance, and agility exercises, was able to increase femoral neck BMD in older women. Interestingly, Kemmler et al. (2010) showed that 18 months of high-intensity training (combined aerobic and strength exercises, 4 days/week) increased lumbar spine and femoral neck BMD in women (68.9 y.o.) compared with a general wellness program. Compared with the present research, Kemmler et al. (2010) and Marques et al. (2011) used a longer training period and combined strength with aerobic training in their MT programs, which appears to be the most effective stimulus to increase or prevent skeletal mass loss during the aging process (Gomez-Cabello et al., 2012). Moreover, in the current study, the lack of statistically significant improvement in bone mass may be attributable to our higher baseline levels. The skeleton’s response to training seems to be dependent on bone mass values (Slatkovska et al., 2010) and greater increments could be obtained if the initial values are lower (Lai et al., 2013; Mosti et al., 2013). Consequently, the studies that include women with different conditions (i.e., with healthy BMD values, osteopenia or osteoporosis) may contribute to the variability among their results. Even though further trials are needed to study MT protocols that specifically focus on the most predisposed regions of fracture, such as the spine and femoral neck, the variety of training programs may also explain the discrepancies observed between studies.

### Comparison

In the current research, there were no statistically significant post-interventions differences in bone parameters between experimental groups. Hence, and in contrast with the initial hypotheses, the claim cannot be made that MT is better than WBV (or vice versa). However, the pre-post changes identified in bone mass at the lumbar spine following WBV and the clinically important pre-post improvements in BMC after MT could be a promising result. Based on these findings, it can be inferred that both training protocols may be a suitable option to enhance bone health in postmenopausal women. In effect, and according to the mechanostat theory (Frost, 1987), it is plausible that both training conditions induced a sufficient mechanical stimuli that caused bone structure to adapt as a response to the strain imposed by physiological loads (muscular contractions) (Schoenau, 2005).

### Limitations

There are some limitations to the present study that should be addressed. The small sample size and the high experimental withdrawals possibly influenced the research outcomes (Faber and Fonseca, 2014) and prevented us from identifying statistically significant differences between groups. The high drop outs rate may be explained by the duration of the intervention (i.e., 24 weeks) and by the high mandatory attendance rate (90%, a minimum of 65/72 sessions had to be completed). For this reason, it was not possible to maintain the initial sample from week 1 to week 24 due to several motives not related to the intervention program, as depicted in the flow diagram. Furthermore, intention-to-treat (ITT) analyses were not included in the data extraction with regards to participants who dropped out during the intervention period. The lack of data concerning each subject’s calcium and vitamin D intake is another limitation. Although volunteers were instructed to maintain their normal daily routines and eating habits, we cannot exclude that possible changes in their dietary intake occurred and affected bone metabolism. The fact that MT interventions have used different training methods makes it difficult to compare across them and could explain the inconsistency of the findings.

## Conclusion and Practical Applications

This study concluded that 24 weeks of supervised exercise programs may improve bone health in postmenopausal women. Our results are encouraging as clinically important increases in lumbar spine bone mass were observed with WBV (BMD and BMC) and MT (BMC), thus, demonstrating that both training programs may counteract the rapid loss of bone mass after the cessation of menstruation. As there were no differences between groups, it is not possible to determine which training protocol produces greater adaptations. Although these training methods seem to be efficacious (the women who perform them, benefit from them), the large dropout rates suggest that, if not applied on an intrinsically motivated group of participants, WBV and MT efficacy (successful application on a large population) may be affected.

Concerning its practical application, WBV may be a promising method to apply in postmenopausal women, as benefits can be achieved with shorter duration sessions when compared to other training modes. Our results suggest that this protocol provides an intense stimulus to improve bone mass in the lumbar spine and support the existing WBV guidelines: (1) supervised protocols should be performed 3 times per week, with high vibration frequency (20–45 Hz) and low magnitude (<1 g), or low frequency (<20 Hz) and high vibration magnitude (1–3 g) (Fratini et al., 2016; Oliveira et al., 2016); and (2) at least 24 weeks are needed to elicit positive adaptations (Oliveira et al., 2016). With respect to MT interventions, professionals should take into consideration that the characteristics of the program used in this study may not be as effective in increasing or preventing skeletal mass loss during the aging process in women as the guidelines reported elsewhere (Marin-Cascales et al., 2017a): a combination of resistance training, aerobic, and high-impact exercises (jogging, jumping, and stepping). Nevertheless, it is worth noting that the protocol used in this research may be performed without specific resistance training facilities or equipment.

Further research is needed to determine what is the optimal WBV and MT dose-response to elicit bone adaptations at the lumbar spine and femoral neck in postmenopausal women, as there are the several methodological differences in the programs currently found in the literature. In order to generate higher osteogenic effects on bone mass and prevent fractures in this population, WBV and MT characteristics such as training frequency, volume, working time and intensity still need clarification.

## Data Availability

The datasets generated for this study are available on request to the corresponding author.

## Ethics Statement

The study protocol was approved by the Human Ethics Committee of the Catholic University of Murcia (UCAM), number CE111212.

## Author Contributions

EM-C participated in the design and coordination of the study, helped to determine training protocols, supervised the training programs, collected and interpreted the data, carried out the literature search, and drafted the manuscript. JR-A participated in the design of the study, helped to determine training protocols, carried out the statistical analysis, interpreted the data, and drafted the manuscript. PA participated in the design of the study, helped to determine training protocols, carried out the tests, and drafted the manuscript. All authors approved the final version of the manuscript, and agreed with the order of presentation of the authors.

## Conflict of Interest Statement

The authors declare that the research was conducted in the absence of any commercial or financial relationships that could be construed as a potential conflict of interest.
